# RNA Polymerase II Transcription Attenuation at the Yeast DNA Repair Gene, *DEF1*, Involves Sen1-Dependent and Polyadenylation Site-Dependent Termination

**DOI:** 10.1534/g3.118.200072

**Published:** 2018-04-23

**Authors:** Courtney Whalen, Christine Tuohy, Thomas Tallo, James W. Kaufman, Claire Moore, Jason N. Kuehner

**Affiliations:** * Department of Nutritional Sciences, College of Health and Human Development, The Pennsylvania State University, University Park, PA 16802; † Department of Microbiology and Physiological Systems, University of Massachusetts Medical School, Worcester, MA 01605; ‡ Division of Infectious Diseases, Boston Children’s Hospital, Harvard Medical School, Boston, MA 02115; § Department of Biology, Emmanuel College, Boston, MA 02115; ** Department of Developmental, Molecular, and Chemical Biology, Tufts University School of Medicine, Boston, MA 02111

**Keywords:** RNA polymerase II termination, attenuation, Def1, cleavage and polyadenylation complex (CPF-CF), Nrd1/Nab3/Sen1 complex (NNS)

## Abstract

Termination of RNA Polymerase II (Pol II) activity serves a vital cellular role by separating ubiquitous transcription units and influencing RNA fate and function. In the yeast *Saccharomyces cerevisiae*, Pol II termination is carried out by cleavage and polyadenylation factor (CPF-CF) and Nrd1-Nab3-Sen1 (NNS) complexes, which operate primarily at mRNA and non-coding RNA genes, respectively. Premature Pol II termination (attenuation) contributes to gene regulation, but there is limited knowledge of its prevalence and biological significance. In particular, it is unclear how much crosstalk occurs between CPF-CF and NNS complexes and how Pol II attenuation is modulated during stress adaptation. In this study, we have identified an attenuator in the *DEF1* DNA repair gene, which includes a portion of the 5′-untranslated region (UTR) and upstream open reading frame (ORF). Using a plasmid-based reporter gene system, we conducted a genetic screen of 14 termination mutants and their ability to confer Pol II read-through defects. The *DEF1* attenuator behaved as a hybrid terminator, relying heavily on CPF-CF and Sen1 but without Nrd1 and Nab3 involvement. Our genetic selection identified 22 *cis*-acting point mutations that clustered into four regions, including a polyadenylation site efficiency element that genetically interacts with its cognate binding-protein Hrp1. Outside of the reporter gene context, a *DEF1* attenuator mutant increased mRNA and protein expression, exacerbating the toxicity of a constitutively active Def1 protein. Overall, our data support a biologically significant role for transcription attenuation in regulating *DEF1* expression, which can be modulated during the DNA damage response.

RNA Polymerase II (Pol II) transcribes a wide assortment of transcripts in eukaryotes, including all protein-coding mRNAs and most non-coding RNAs. The high density of Pol II across the genome must be confined to prevent interference with surrounding protein:DNA transactions (Jensen *et al.* 2013). Genomic partitioning is achieved in part by transcription termination, which releases Pol II and its RNA product from the DNA template. The biological significance of termination is not limited to defining transcriptional boundaries. Due to its connection with 3′-end processing, stability, and export, Pol II termination has the ability to influence RNA fate and function (Kuehner *et al.* 2011; Mischo and Proudfoot 2013; Arndt and Reines 2015; Porrua and Libri 2015). In addition, there is an expanding collection of genes regulated by premature transcription termination (*i.e.*, attenuation), some of which exhibit altered expression during cell metabolism and stress. The significance of Pol II termination has also been revealed in human pathology, including its roles in HIV latency, herpes viral infection, and renal cell carcinoma (Natarajan *et al.* 2013; Rutkowski *et al.* 2015; Grosso *et al.* 2015; Loya and Reines 2016).

Pol II termination is best understood in the model eukaryote *S. cerevisiae*, which is especially dependent on the process due to its compact genome (Goffeau *et al.* 1996). In yeast, Pol II termination occurs via two major pathways: cleavage and polyadenylation factor (CPF-CF) termination and Nrd1-Nab3-Sen1 (NNS) termination (Kuehner *et al.* 2011; Mischo and Proudfoot 2013; Arndt and Reines 2015; Porrua and Libri 2015). Pol II termination of most mRNAs occurs via CPF-CF termination, during which recognition of a polyadenylation (pA) site contributes to 3′-end processing and Pol II eviction, perhaps via allosteric changes in the elongation complex. Pol II termination of most noncoding RNAs occurs via NNS termination. Noncoding RNAs, including some small nuclear RNAs (snRNAs) and small nucleolar RNAs (snoRNAs), can be processed by endonucleolytic cleavage and exonucleolytic trimming, resulting in stable and abundant products (Bernstein and Toth 2012). Noncoding RNAs can also behave as cryptic unstable transcripts (CUTs) due to NNS-mediated oligoadenylation via the TRAMP complex and exosome-mediated degradation (Schmidt and Butler 2013; Tudek *et al.* 2014). The selection of CPF-CF *vs.* NNS termination depends on RNA sequence elements and protein factors associated with the Pol II Rpb1 C-terminal domain (CTD), which is modified in accordance with Pol II distance from a transcription start site (TSS) (Rondon *et al.* 2008). There is evidence to support direct roles for a Pol II CTD-binding protein (Pcf11), an RNA exonuclease (Rat1), and an RNA/DNA helicase (Sen1) in the actual Pol II release step, but the termination mechanism remains unresolved (Kuehner *et al.* 2011; Mischo and Proudfoot 2013; Arndt and Reines 2015; Porrua and Libri 2015).

While CPF-CF and NNS termination pathways exhibit distinct behavior, they display substantial overlap among factor requirements, and they may have evolved independently to recognize highly similar sequence elements (Porrua *et al.* 2012). NNS termination typically occurs < 1 kb from a TSS due to Nrd1-mediated recognition of Ser5-phosporylated CTD, which is predominant when Pol II is proximal to the promoter (Rondon *et al.* 2008). CPF-CF termination typically occurs > 1 kb from a TSS due to Pcf11-mediated recognition of Ser2-phosphorylated CTD, which is predominant when Pol II is distal to the promoter. However, exceptions have been identified that circumvent these trends. NNS terminators can be recognized outside of their normal context and distance threshold, in some cases acting as a fail-safe mechanism to prevent transcription interference (Steinmetz *et al.* 2006a; Gudipati *et al.* 2008; Ghazal *et al.* 2009; Rondón *et al.* 2009; Porrua *et al.* 2012). Likewise, NNS terminators can be dependent on CPF-CF components (Fatica *et al.* 2000; Morlando *et al.* 2002; Dheur *et al.* 2003; Kim *et al.* 2006; Pearson and Moore 2014). The basis of Pol II flexibility in terminator recognition remains unclear, as does the extent of crosstalk and function between CPF-CF and NNS components.

In addition to Pol II termination occurring downstream of genes, premature Pol II termination (attenuation) can regulate mRNA gene expression. Attenuation has long been recognized as a widespread mechanism of bacterial gene regulation (Naville and Gautheret 2010), but the extent of its activity and biological significance has been studied more recently in eukaryotes (Colin *et al.* 2011; Contreras *et al.* 2013). Genome-wide analysis of CUTs and NNS factors suggest that 5–10% of yeast mRNA genes may be regulated by attenuation (Neil *et al.* 2009; Webb *et al.* 2014). The *NRD1* gene was identified as an early target of attenuation in yeast, whereby Nrd1 autoregulates its mRNA expression as part of the NNS termination complex (Arigo *et al.* 2006). Similar autoregulatory schemes appear to operate for RNA processing factor genes *HRP1* and *PCF11* (Kuehner and Brow 2008; Creamer *et al.* 2011). Attenuator recognition and bypass has been linked to changes in cell metabolism and stress response genes, but in most cases the signaling mechanism is unknown. Examples of mRNA gene attenuation targets include *IMD2* and *URA2* (nucleotide biosynthesis), *FKS2* (cell wall damage), *CLN3* (glucose starvation), *GPH1* (glycogen metabolism), and *GLT1* (nitrogen metabolism) (Jenks *et al.* 2008; Kuehner and Brow 2008; Thiebaut *et al.* 2008; Kwapisz *et al.* 2008; Kim and Levin 2011; Darby *et al.* 2012; Chen *et al.* 2017; Merran and Corden 2017).

In a genome-wide study of yeast polyadenylation site usage, our labs identified *DEF1* as a gene that could be regulated by attenuation (Graber *et al.* 2013). *DEF1* encodes a protein needed for degradation of Pol II stalled at UV-induced lesions, thus providing nucleotide excision repair factors with access to DNA (Woudstra *et al.* 2002). *DEF1* function is regulated at the level of post-translational control, during which UV damage induces Def1 protein processing and nuclear accumulation (Wilson *et al.* 2013; David 2013). In this study, we confirmed that the *DEF1* promoter-proximal pA site is sufficient to behave as a *bona fide* attenuator, supporting an unexpected mechanism for *DEF1* regulation. Using a plasmid-based reporter system, we identified several *cis*- and *trans*-acting requirements, including a putative Hrp1-binding site and factors in both CPF-CF and NNS terminator pathways. Despite its promoter-proximal location, the *DEF1* attenuator behaved most similarly to a hybrid CPF-CF-Sen1 terminator rather than a traditional NNS terminator. To demonstrate biological significance, we have shown that an attenuator mutation results in *DEF1* overexpression, enhancing the toxicity of a constitutively active Def1 protein. To our knowledge, this is the first example of attenuator regulation being linked to a DNA damage response gene.

## MATERIALS AND METHODS

### Construction of pGAC24-CUP1 and pGAC24-lacZ reporter gene plasmids

PCR using Q5 Hot Start High-Fidelity 2X Master Mix (NEB) was performed to amplify the *DEF1* attenuator region (-187 to +93, relative to the +1 ATG) from yeast strain BY4742 genomic DNA and cloned into the *Xho*I restriction site of the pGAC24-No Term-*CUP1* (no terminator) vector (Lesser and Guthrie 1993), which had been treated with Antarctic phosphatase (NEB) to prevent religation of linearized plasmid. The ligation was performed overnight at 16° using T4 DNA ligase (NEB) prior to transformation into 5-alpha Competent *E. coli* cells (NEB) to create pGAC24-*DEF1-CUP1*.

PCR using Q5 Hot Start High-Fidelity 2X Master Mix (NEB) was performed to amplify relevant fragments from plasmids pGAC24-*DEF1-CUP1* (GAP promoter, *ACT1* intron with *DEF1* attenuator, *ACT1* exons, *AMP*^R^ gene, *LEU2* gene) and p903 (*lacZ* ORF) (Guarente and Mason 1983). The two PCR products were assembled to create the pGAC24-*DEF1-lacZ* vector using a Gibson Assembly Cloning Kit (NEB). The pGAC24-No Term-*lacZ* plasmid (no terminator) was generated by *Xho*I restriction digestion of pGAC24-*DEF1-lacZ*, separation of backbone from insert using a ZymoClean Gel DNA recovery kit (Zymo Research), and religation of backbone lacking insert. The *CYC1* terminator (+448 to +528 relative to +1 ATG start codon) and *SNR13* terminator (+125 to +232 relative to +1 TSS) were amplified by PCR and cloned into the *Xho*I site of pGAC24-no Term-*lacZ*. A pGAC24-*DEF1-lacZ* (*HIS3*) reporter was generated using Gibson cloning to swap in the *HIS3* selectable marker in place of *LEU2*, and the no Term, *CYC1*, and *SNR13* reporters were generated by PCR cloning and *Xho*I digestion as described above. Transformation of reporter plasmids into yeast strains was performed using a standard lithium acetate method.

### Construction of pRS426-DEF1 plasmid and A-1G and C1590A mutants

PCR using Q5 Hot Start High-Fidelity 2X Master Mix (NEB) was performed to amplify the *DEF1* promoter, 5′-UTR, ORF, and 3′-UTR (-428 to +2596 relative to the +1 ATG) from yeast strain BY4742 genomic DNA and cloned into the *Not*I restriction site of the pRS426 (*URA3*) vector. The A-1G and C1590A point mutations were generated using a Quik-Change Lightning Site-Directed Mutagenesis Kit (Agilent).

### Construction of pRS314-HRP1 plasmid and the hrp1-5 mutant

PCR using Q5 Hot Start High-Fidelity 2X Master Mix (NEB) was performed to amplify the *HRP1* promoter, 5′-UTR, ORF, and 3′-UTR (-500 to +1848 relative to the +1 ATG) from yeast strain BY4742 genomic DNA and cloned into the *Not*I/*Xho*I restriction site of the pRS314 (*TRP1*) vector. The L205S point mutation (*hrp1-5*) was generated using a Quik-Change Lightning Site-Directed Mutagenesis Kit (Agilent).

### Spot Test Assays

Yeast bearing the reporter plasmids of interest were grown overnight in a shaking incubator (at 25° or 30°) in appropriate selective liquid media. The absorbance at OD_600_ was measured for each strain using a spectrophotometer (Implen Diluphotometer) and cultures were diluted to OD_600_ = 1.0. Each diluted culture (200 μL) was transferred to a 96-well plate, followed by 10-fold serial dilutions (x4) into adjacent wells. All samples were mixed and spotted using a 96-well replica plater (Sigma) on appropriate plate media. The plates were air-dried and incubated for 3-7 days.

### Yeast Doubling-Time Growth Assays

The BY4742 *def1Δ* strain bearing the pRS426-*DEF1* plasmids of interest were grown overnight in a shaking 30° incubator in -Ura media. The absorbance at OD_600_ was measured for each strain using a spectrophotometer (Implen Diluphotometer) and cultures were diluted to OD_600_ = 0.15. Each diluted culture was grown for 4 hr at 30° (recovery period) and then split into separate cultures, with half of the culture shifted to 39° and the other half remaining at 30° for 2.5 hr. The absorbance at OD_600_ was measured every half hour, and doubling times were calculated using the following formula: Doubling time = (Time Duration x log(2)) / (log (Final Concentration – log (Initial Concentration))).

### Yeast β-galactosidase Microplate Plate Assay

Yeast bearing the reporter plasmids of interest were grown overnight in a shaking incubator (30° for most strains or 25° for *nab3-11 and hrp1-5*) in appropriate selective liquid media. The cultures were diluted 10-fold and absorbance at OD_600_ was measured for each strain using a plate reader (SpectraMax 190). Cultures were diluted to OD_600_ = 0.15, grown at 30° for 2 hr (recovery period), and shifted to 37° for 2 hr (non-permissive temperature). Each strain (200 μL) was aliquoted into a 96-well plate and media containing no cells served as an instrument blank. Following measurement of absorbance at OD_600_, cell lysis was performed and reporter enzyme activity was measured using the Yeast β-Galactosidase Assay Kit according to manufacturer’s instructions (Thermo Scientific). The assay kit working solution was prepared and added separately to the 96-well plate in 100 µL aliquots. The absorbance at OD_420_ was measured every minute for 60 min. to collect a kinetic reaction rate and slope. Slopes were gathered using a kinetic reaction window in a linear range with strong R^2^ value. Relative β-galactosidase activity was calculated using the equation: ((OD_420_ Slope) ÷ (0.1 mL) x OD_600_) = βeta-galactosidase activity (Thibodeau *et al.* 2004).

### Mutagenesis of the Attenuator

Mutagenesis of the *DEF1* attenuator was carried out by error-prone PCR and *in-vivo* homologous recombination (Muhlrad *et al.* 1992). Primers complementary to pGAC24-*DEF1-CUP1* sequences were used to direct amplification by Taq DNA polymerase (NEB) of a 635 bp fragment containing the *DEF1* 5′-UTR and upstream ORF. PCR amplification was performed for 40 rounds, purified, and repeated for an additional 40 rounds, relying upon the normal error rate of Taq DNA polymerase for the introduction of random mutations. pGAC24-*DEF1-CUP1* was digested with *Bam*HI and *Xho*I, creating a gap of 447 bp flanked by 110 and 78 nt of complementarity to the PCR product, and cotransformed with the PCR product into the 46α yeast strain. After selection for transformants on -Leu plates, colonies were replica plated onto -Leu plates containing 0.4 mM CuSO_4_. Plasmids were recovered from copper-resistant colonies and retransformed into yeast to confirm that the copper-resistant phenotype was linked to the plasmid prior to sequencing.

### Reverse transcriptase (RT)-PCR

Total RNA was purified from yeast cells by measuring the absorbance at OD_600_ of the desired culture and harvesting 1.5 ODs of cells. The Master Pure Yeast RNA Purification Kit (Epicentre) was used to purify total RNA (including 1 hr DNase I treatment), and RNA quality and yield was determined using a NanoDrop spectrophotometer (ThermoFisher). To convert the purified RNA to cDNA, RT-PCR was performed using a OneTaq RT-PCR kit (NEB). Pairwise reactions were set up including 1 ug of total RNA and random primer (mix of hexamer and d(T)_23_VN primers). A negative control received no reverse transcriptase (-RT) to ensure that the final signal was RNA-dependent and not derived from chromosomal DNA template. Amplification of *CUP1*, *lacZ*, *DEF1*, and 18S RNA was performed using OneTaq DNA polymerase in a 25 μL reaction with 2 μL of diluted cDNA template, as directed by the manufacturer. The amplification cycle number ranged from 12-25 cycles depending on the linearity and intensity of the PCR product signal. The PCR products (10 μL) were loaded into a 2% agarose gel stained with SYBR Safe (Invitrogen). The gel band intensity and ratios of total, read-through, and attenuated transcripts were measured using the Gel Doc EZ System (BioRad) and ImageStudio software (LICOR).

### Western blot analysis of Def1 protein levels

The BY4741 *def1*Δ strains containing pRS426-*DEF1* or the *def1* mutants were grown overnight at 30°, diluted back to OD_600_ = 0.4, grown for 4 hr in a 30° shaking incubator (recovery), and shifted to a 39° shaking incubator (non-permissive temperature) for 0, 1, or 2 hr. The cells were harvested (10 OD units), washed in 20% TCA, and stored at -20°. Whole cell protein extracts were prepared using a TCA method (Keogh *et al.* 2006). Briefly, cell pellets were resuspended in 250 μL of 20% TCA and lysed using glass beads and a vortexer in a 4° room (3 × 1 min) with 1 min pauses on ice between runs. The supernatant was transferred to a new tube using a gel-loading tip to avoid the beads. 700 μL of 5% TCA was added to the supernatant (1.25 mL final) and inverted to mix. The sample was microcentrifuged at 13,300 RPM for 10 min at 4°. The supernatant was discarded and the pellet was washed with 750 μL of 100% ice-cold ethanol. The wash buffer was discarded, and the pellet was resuspended in 40 μL of 1 M Tris Cl, pH 8.0. An additional 80 μL of 2X SDS reducing sample buffer (66 mM Tris-HCl pH 6.8, 26% (w/v) glycerol, 2.1% SDS, 0.01% bromophenol blue) was added, and the sample was heated to 95° for 5 min, microcentrifuged at top speed for 5 min., and the supernatant was used for Western blot analysis. Western blots were incubated with rabbit polyclonal anti-Def1 antibody (1:5,000; kind gift of Svejstrup lab) and mouse anti-β actin antibody (1:5,000, Abcam) prior to incubation with an anti-rabbit or anti-mouse HRP-conjugated secondary antibody (1:15,000; Jackson Immunoresearch). Target proteins were detected via chemiluminescence using Clarity ECL Western blotting substrate (BioRad) and a C-Digit blot scanner with Image Studio software (LICOR).

### Data Availability

Strains, plasmids, and primer sequences are available upon request. The authors affirm that all data necessary for confirming the conclusions of the article are present within the article, figures, and tables. Supplemental material available at Figshare: https://doi.org/10.25387/g3.6167696.

## RESULTS

### DEF1 promoter-proximal region resembles a transcriptional attenuator

**Figure 1:**
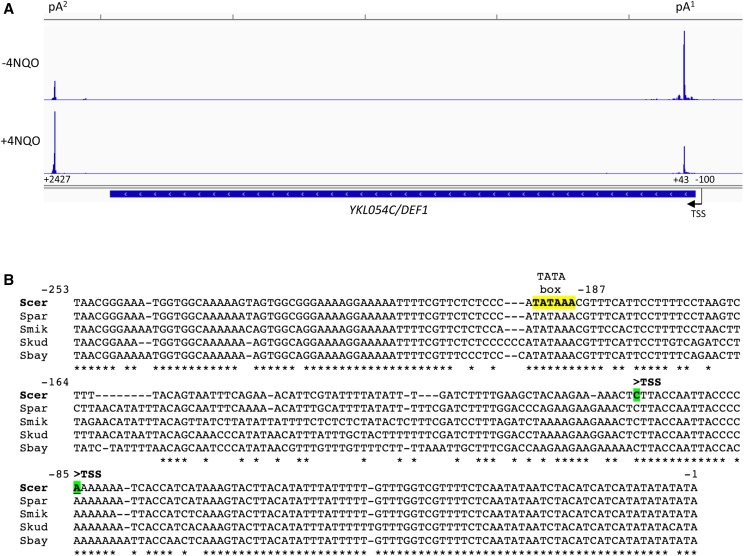
*DEF1* promoter-proximal region resembles a transcriptional attenuator. (A) Direct RNA sequence tags were mapped to the *DEF1* region of the *S. cerevisiae* genome (Integrative Genomics Viewer) in strains grown -/+ the UV-mimetic drug 4-NQO (Graber *et al.*, 2013). In this image, the *DEF1* gene is transcribed to the left (TSS indicated by arrow), with vertical bars indicating the number of pA site reads corresponding to each position. Positions of major TSS and pA sites are numbered relative to the *DEF1* +1 start codon (ATG). Tick marks at the top of the IGV image correspond to 500 bp segments. (B) Alignment of *DEF1* promoter / 5′-UTR in five species of the genus *Saccharomyce*s. Sequences were obtained using *Saccharomyces* Genome Database (SGD) Fungal alignment. All non-template strand sequences between -253 and -1 relative to the *DEF1* start codon are shown, with invariant nucleotides indicated with asterisks. The putative TATA box (yellow) and mapped TSS (green) (Zhang *et al.* 2005; Nagalakshmi *et al.* 2008) are also indicated.

Our labs have previously identified *DEF1* as a gene with both promoter-proximal (pA^1^) and promoter-distal (pA^2^) polyadenylation sites (Graber *et al.* 2013). This arrangement of signals for *DEF1* 3′-end processing results in multiple mRNA isoforms due to alternative pA site usage. In the absence of stress ∼70% of *DEF1* transcripts terminate near pA^1^ (< 50 nt downstream of the start codon), resulting in a mixture of short RNAs (∼150 nt) that are not capable of being translated into Def1 protein. Upon exposure to the DNA damaging agent 4-NQO, pA^1^ usage is reduced to ∼30%, resulting in ∼70% of polyadenylation occurring at pA^2^, which is ∼2.5 kb downstream of the TSS (Figure 1A). The switch in pA usage likely contributes to the ∼twofold increase in full-length *DEF1* mRNA produced upon exposure to 4-NQO. Aside from the observed changes in pA/terminator usage, *DEF1* bears additional hallmarks of attenuator regulation. The promoter-proximal *DEF1* transcripts consist of both a polyadenylated fraction and a CUT fraction (Neil *et al.* 2009; Ozsolak *et al.* 2010; Graber *et al.* 2013), which is consistent with the CPF-CF and/or NNS termination pathway operating in this region. In addition, the *DEF1* 5′-UTR is highly conserved among yeast species (Figure 1B), suggesting that the RNA takes on an important regulatory function, as observed for *IMD2* and *HRP1* attenuators (Kuehner and Brow 2008).

### DEF1 promoter-proximal pA site is sufficient to confer Pol II attenuation in a reporter plasmid

To better understand the signals and factors involved in recognition and read-through of the *DEF1* promoter-proximal pA site, we fused the *DEF1* promoter, 5′-UTR, and pA^1^ region (*pDEF1-pA^1^*) from -253 to +93 (relative to +1 start codon) to the *CUP1* reporter gene, which confers resistance to copper-containing media in a *cup1Δ* strain background (Kuehner and Brow 2008). The *pDEF1-pA^1^-CUP1* strain was copper sensitive but failed to exhibit copper resistance in any of the termination factor mutants tested (data not shown). The unexpected behavior of *pDEF1-pA^1^-CUP1* was likely due to misfolding of the Def1-Cup1 fusion protein upon addition of *DEF1* encoded amino acids (+1 to +93). A fusion of *CUP1* to the *DEF1* promoter and 5′-UTR in the absence of the upstream *DEF1* ORF (-253 to -1) resulted in only modest sensitivity on 0.8 mM copper (data not shown).

**Figure 2:**
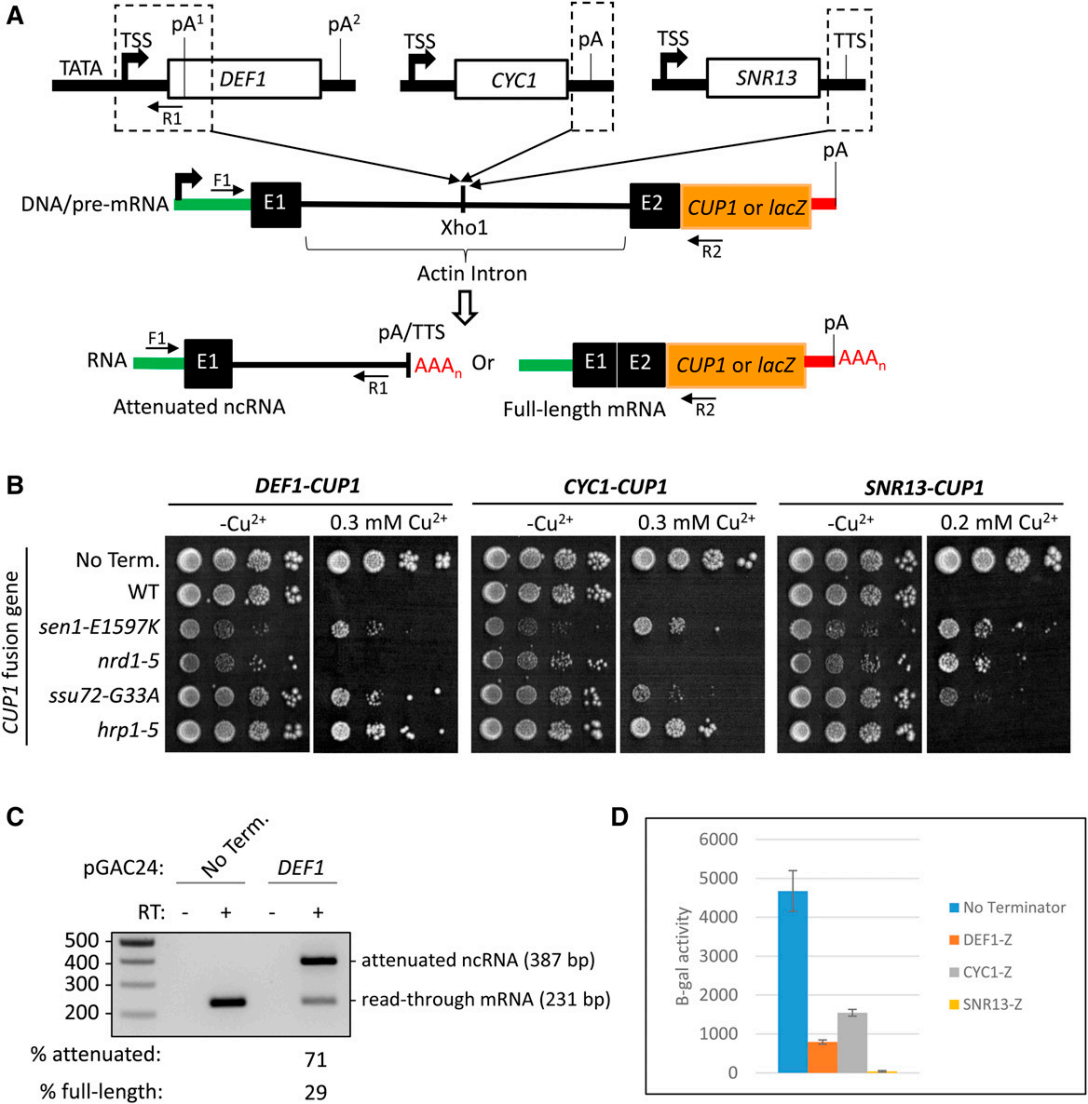
*DEF1* promoter-proximal pA site (pA^1^) is sufficient to confer Pol II transcription attenuation in a *CUP1*/lacZ reporter assay. (A) Schematic of CUP1/lacZ reporter gene used to measure transcription termination (not to scale). The pGAC24 plasmid contains the actin exon(E1)-intron-exon(E2) fused to a *CUP1* or lacZ reporter gene. The promoter-proximal *DEF1* pA site, *CYC1* pA site, or *SNR13* transcription termination site were inserted within the intron. The *DEF1* attenuator includes the 5′-UTR and upstream ORF but not the consensus TATA box promoter element. In the absence of a pA/terminator insert (No Term.), full-length mRNA production confers copper-resistance and high β-galactosidase activity. In the presence of a pA/terminator insert, attenuated non-coding RNA (ncRNA) production confers copper-sensitivity and low β-galactosidase activity. *Trans*-acting mutants that prevent pA/terminator recognition promote copper-resistance and higher β-galactosidase activity. (B) *DEF1* pA^1^ site confers copper-sensitivity in a *DEF1-CUP1* reporter, and *sen1*, *ssu72*, and *hrp1* mutants confer copper-resistance. (C) *DEF1* pA^1^ site reduces expression of *CUP1* mRNA due to accumulation of attenuated ncRNA. Note that based on the RT-PCR primer locations (F1, R1, and R2 in panel A), the RT-PCR product from spliced mRNA (231 bp) is shorter than the PCR product from attenuated, unspliced transcript (387 bp). The % attenuated *vs.* full-length was determined by adding up signal intensities for both bands and determining the relative ratio. No Term. = No Terminator. (D) *DEF1* pA^1^ site reduces expression of a lacZ reporter similarly to known transcription terminators from *CYC1* and *SNR13*. β-galactosidase activity was measured following cell lysis and incubation with ONPG substrate, using absorption at OD_600_ for cell density and OD_420_ for product production. Experiments were performed in biological triplicate, and errors bars show standard deviation.

To test the activity of the *DEF1* pA^1^ site in a context where the ORF region is not translated into protein, we cloned the *DEF1* attenuator into the intron of the pGAC24-*CUP1* reporter gene plasmid (Figure 2A) (Lesser and Guthrie 1993; Steinmetz and Brow 1996). The *DEF1* attenuator included the *DEF1* 5′-UTR and the pA^1^ within the upstream ORF (-187 to +93) but not the consensus TATA box promoter element. In the absence of a transcriptional terminator insert (No Term.), the *cup1∆* strain was copper-resistant as expected (Figure 2B). Insertion of the *DEF1* pA^1^ into the pGAC24 reporter was sufficient to confer copper sensitivity to 0.3 mM copper (Figure 2B). The *DEF1* pA^1^ insert resulted in predominant production (71%) of short RT-PCR products, consistent with transcription attenuation (Figure 2C). Mutations in the NNS protein Sen1 and the CPF-CF proteins Ssu72 and Hrp1 conferred *DEF1-CUP1* resistance to 0.3 mM copper, while a mutation in the NNS Nrd1 protein had no effect (Figure 2B). For comparison, we analyzed transcription termination activity from the mRNA gene *CYC1*, which contains a hybrid CPF-CF-NNS-dependent terminator (+448 to +528 relative to +1 ATG start codon), and the snRNA gene *SNR13*, which contains an NNS-dependent terminator (+125 to +232 relative to +1 TSS). The *CYC1* gene contains a traditional mRNA 3′-end processing site but is somewhat NNS-dependent, at least in part because it is a short gene containing a pA site located <1 kb from the promoter (Steinmetz *et al.* 2006a). The *sen1*, *ssu72*, and *hrp1* mutants conferred resistance to 0.3 mM copper for *CYC1-CUP1*, and the *sen1*, *nrd1*, and *ssu72* mutants conferred resistance to 0.2 mM copper for *SNR13-CUP1* (Figure 2B). These data for the control terminators were mostly consistent with previous reports (Steinmetz *et al.* 2001; Steinmetz and Brow 2003; Steinmetz *et al.* 2006a; Chen *et al.* 2017).

To extend the versatility of the reporter gene and improve quantitation of read-through defects, we replaced the *CUP1* gene with the *lacZ* gene, which allows β-galactosidase activity to be used as a readout of transcriptional activity. In addition, the *lacZ* reporter in the pGAC24 plasmid is under control of a constitutive promoter instead of a previously described *GAL*-inducible *lacZ* reporter (Hyman *et al.* 1991). The ability to maintain the pGAC24-*DEF1-lacZ* reporter in selective glucose media avoids complications arising from the galactose-sensitivity of some termination mutants (data not shown). In the absence of a terminator (No Term.), the reporter produced ∼4,500 units of β-gal activity (Figure 2D). The *DEF1-lacZ* reporter produced ∼800 β-gal units, which is a >fivefold reduction compared to the no terminator control. The *CYC1-lacZ* reporter produced ∼1500 β-gal units (threefold reduction), and the *SNR13-lacZ* reporter exhibited ∼40 units of β-gal activity (120-fold reduction). Overall the *DEF1-lacZ* reporter exhibited an intermediate level of termination activity between the stronger *SNR13* terminator and the weaker *CYC1* terminator (Figure 2D).

### Mutations in both NNS and CPF-CF pathways confer Pol II read-through of DEF1 attenuator

**Figure 3:**
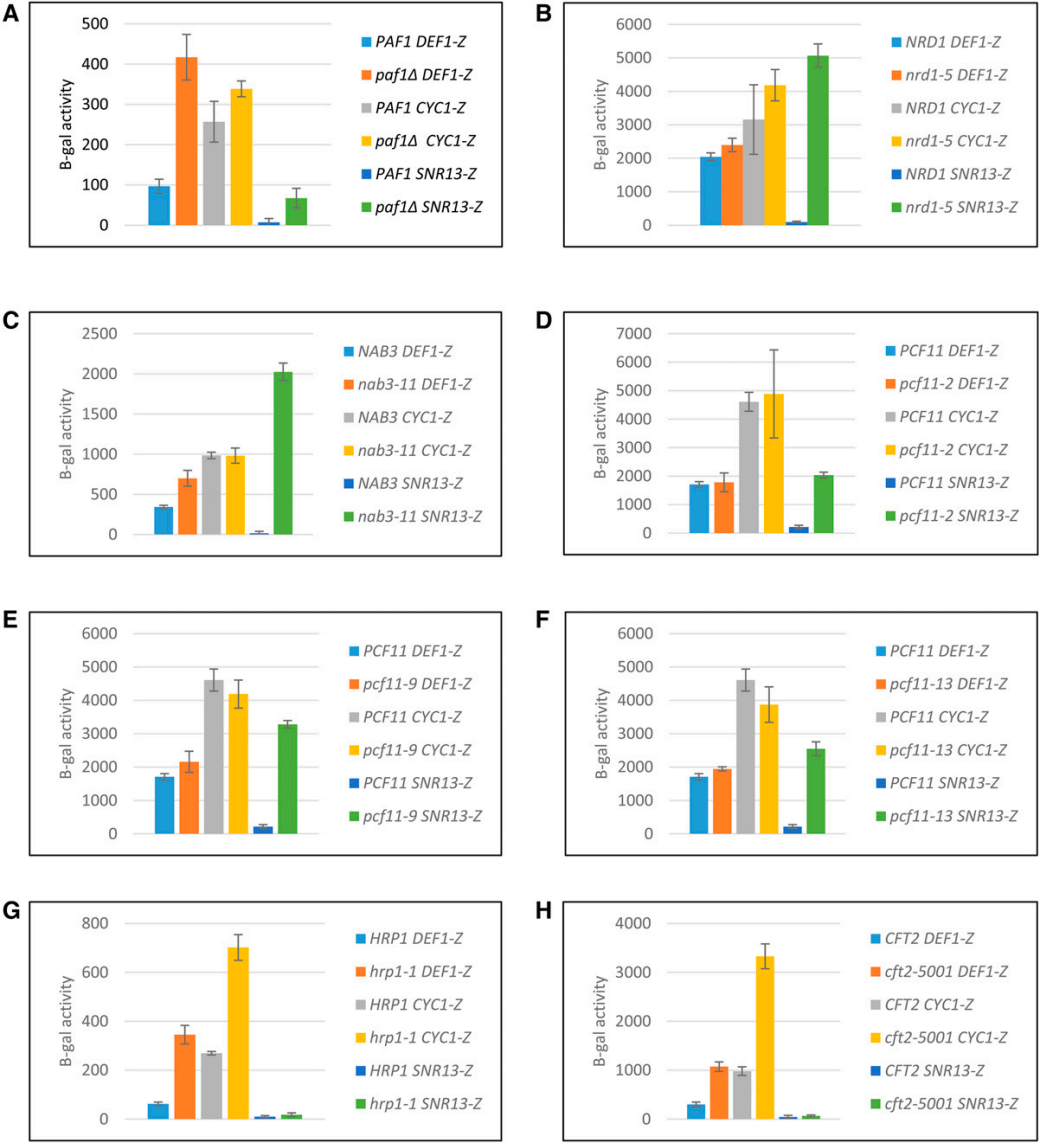
*Trans*-acting mutants *paf1*, *hrp1*, and *cft2* result in Pol II read-through (>twofold) of the *DEF1* attenuator, representing a contribution of both NNS- and CPF-CF-dependent termination activity. (A)-(H) The *DEF1*-lacZ reporter and the control reporters *CYC1*-lacZ (CPF-CF-NNS hybrid terminator) and *SNR13*-lacZ (NNS terminator) were transformed into the indicated strains above. Cultures were grown at permissive temperature (25°C or 30°C) and diluted prior to shifting to 30°C for 2 hr and 37°C (non-permissive) for 2 hr. β-galactosidase activity was measured following cell lysis and incubation with ONPG substrate, using absorption at OD_600_ for cell density and OD_420_ for β-gal production. Experiments were performed in biological triplicate, and errors bars show standard deviation.

**Figure 4:**
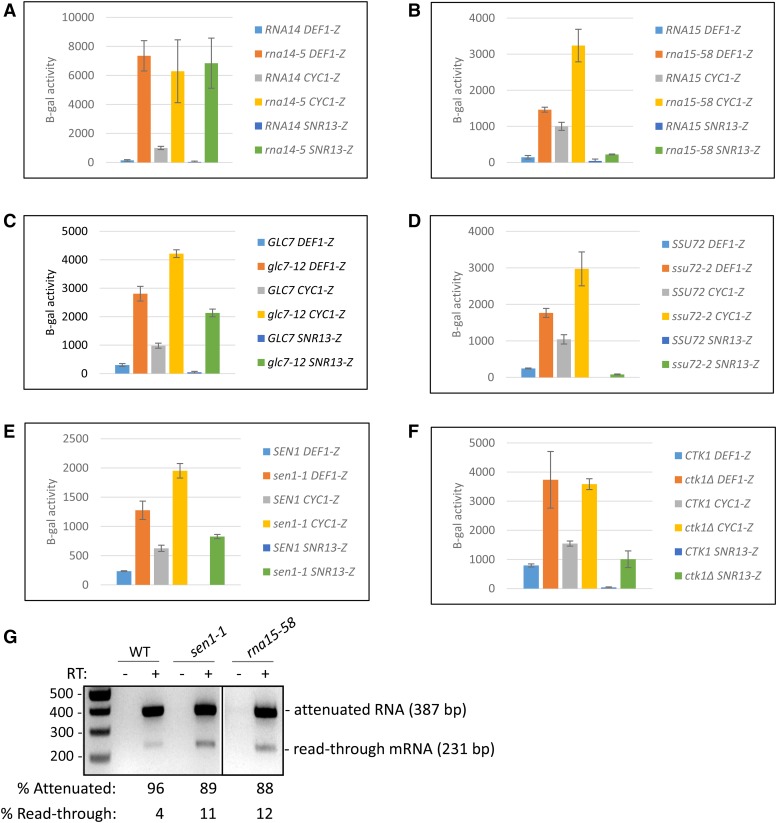
*Trans*-acting mutants *rna14*, *rna15*, *glc7*, *ssu72*, *sen1*, *and ctk1* result in Pol II read-through (>twofold) of the *DEF1* attenuator, representing a contribution of both NNS- and CPF-CF-dependent termination activity. (A)-(F) The *DEF1*-lacZ reporter and the control reporters *CYC1*-lacZ (CPF-CF-NNS hybrid terminator) and *SNR13*-lacZ (NNS terminator) were transformed into the indicated strains above. Cultures were grown at permissive temperature (25°C or 30°C) and diluted prior to shifting to 30°C for 2 hr and 37°C (non-permissive) for 2 hr. β-galactosidase activity was measured following cell lysis and incubation with ONPG substrate, using absorption at OD_600_ for cell density and OD_420_ for β-gal production. Experiments were performed in biological triplicate, and errors bars show standard deviation. (G) Total RNA was collected from indicated strains containing *DEF1-lacZ* grown at 30°C, and attenuated and read-through mRNAs were detected via RT-PCR as done in Fig. 2C. Negative control reactions lacking reverse transcriptase (-RT) were used to ensure that amplification was RNA-dependent.

To investigate the *trans*-acting requirements of *DEF1* attenuation, we measured β-gal reporter activity in response to a variety of Pol II termination mutants. The mutations targeted members of CFI (Pcf11, Rna14, Rna15, Hrp1), CPF (Cft2, Glc7, Ssu72), and NNS (Nrd1, Nab3, Sen1). Our genetic screen also included Ctk1 and Paf1, which promote Pol II modification and recruitment of termination factors (Bowman and Kelly 2014; Van Oss *et al.* 2017). We first classified the effect of mutants on the control terminators to determine their relative impact on NNS *vs.* CPF-CF pathways in our reporter system. Pol II terminator read-through activity of *SNR13-lacZ*, but not *CYC1-lacZ*, was increased (>twofold) by the *paf1Δ*, *nrd1-5*, *nab3-11*, *pcf11-2*, *pcf11-9*, and *pcf11-13* mutants, indicating defects in NNS-dependent termination (Figure 3A-F). In contrast, the reporter activity of *CYC1-lacZ*, but not *SNR13-lacZ*, was increased (>twofold) by the *hrp1-1* and *cft2-5001* mutants, indicating defects in CPF-CF termination (Figure 3G, H). Interestingly, the reporter activity of *DEF1-lacZ* increased (>twofold) in the *paf1Δ*, *hrp1-1*, and *cft2*-1 mutants (Figure 3A, G, H), indicating a dependence on both NNS- and CPF-CF termination pathways for attenuator recognition. We identified an additional class of mutants (*rna14-5*, *rna15-58*, *glc7-12*, *ssu72-2*, *sen1-1*, *ctk1Δ*) that conferred read-through defects for both *SNR13-lacZ* and *CYC1-lacZ* control reporters (Figure 4A-F). All of these mutants likewise increased *DEF1-lacZ* activity, confirming that the *DEF1* attenuator behaves as a hybrid Sen1- and CPF-CF-dependent terminator.

To confirm that the β-gal activity of the reporter genes was due to increased Pol II read-through of the transcriptional terminator, we analyzed RNA from the *DEF1-lacZ* reporter in *sen1-1* and *rna15-58* strains using RT-PCR to detect attenuated and read-through mRNA (Figure 4G). In wild-type strains, the preponderance of RNA (96%) corresponded to attenuated RNA. The higher efficiency of attenuator activity in *DEF1*-lacZ compared to *DEF1-CUP1* (compare Figure 4G to Figure 2C) may reflect differences in Pol II elongation through the reporter genes. The *sen1-1* and *rna15-58* mutants increased the level of read-through mRNA ∼3 fold, which is consistent with the trend observed in the β-gal assay. These data validate the use of β-gal activity from the *DEF1-lacZ* reporter as a proxy for Pol II attenuator recognition and read-through at the RNA level.

**Figure 5:**
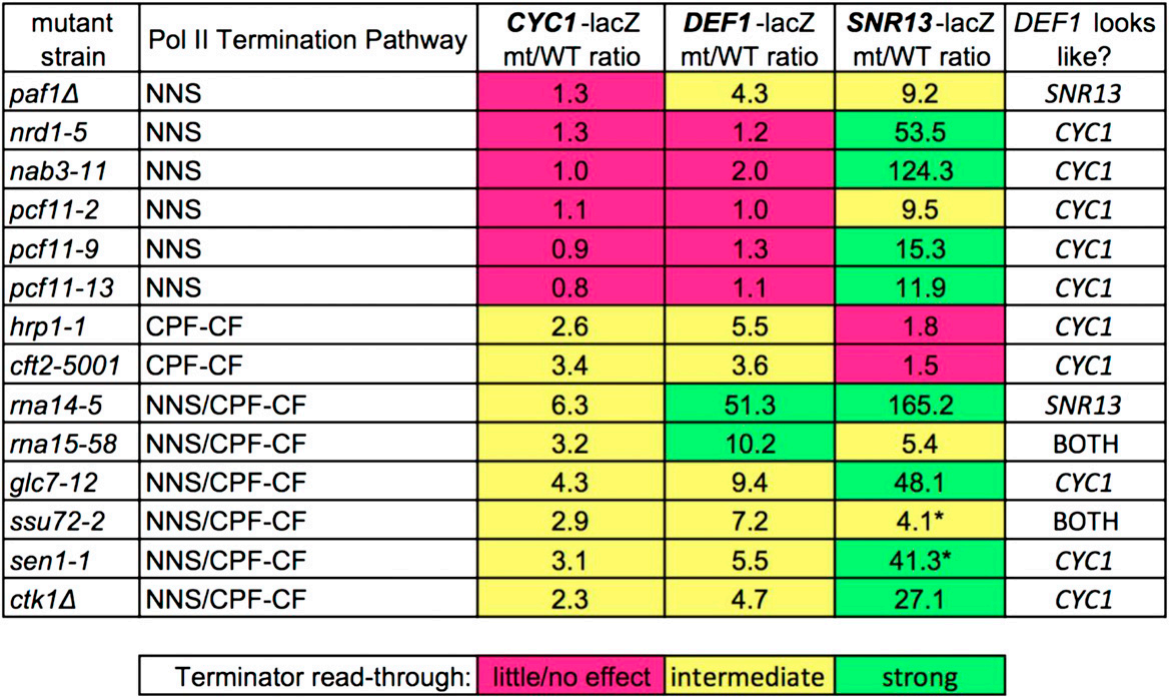
Summary of *trans*-acting mutant effects on *DEF1* attenuator indicates similar behavior to *CYC1* hybrid CPF-CF-NNS terminator. The relative level of terminator read-through in mutant/WT was calculated from lacZ assays in Figs. 3 and 4 and summarized in the table (little/no effect: ≤twofold (pink); intermediate: 2-10 fold (yellow); strong: >10-fold (green). Pol II termination pathways for each factor were assigned based on sensitivity of mutants to a known NNS-dependent terminator (*SNR13*) or hybrid CPF-CF-NNS-dependent terminator (*CYC1*). The asterisk indicates cases in which the kinetic slope of lacZ activity from the WT *SNR13*-lacZ reporter was undetectable, and an approximate value was utilized that was comparable to other experiments (40 B-gal units).

To more quantitatively compare the *DEF1* terminator to control terminators, we created a read-through index to rank the level of β-gal activity in mutant *vs.* wild-type strains. Based on the range of the data, we assigned the mt/WT ratio into 3 categories: little/no effect, ≤twofold; intermediate effect, 2-10 fold; and strong effect, >10-fold (Figure 5). Ranked in order, the mutants most defective for *DEF1* attenuator recognition were *rna14-5* and *rna15-58*, and those with an intermediate effect were *glc7-12*, *ssu72-2*, *hrp1-1*, *sen1-1*, *ctk1Δ*, *paf1Δ* and *cft2-5001*. The *DEF1* attenuator behaved more similarly to the *CYC1* terminator than the *SNR13* terminator, with *DEF1* matching *CYC1* in 10/12 cases that allowed for direct comparison within the index. These results indicate that the *DEF1* attenuator exhibits hybrid characteristics but is more heavily influenced by the CPF-CF termination pathway.

### DEF1 attenuator consists of multiple cis-acting elements spanning the ORF start codon

**Figure 6:**
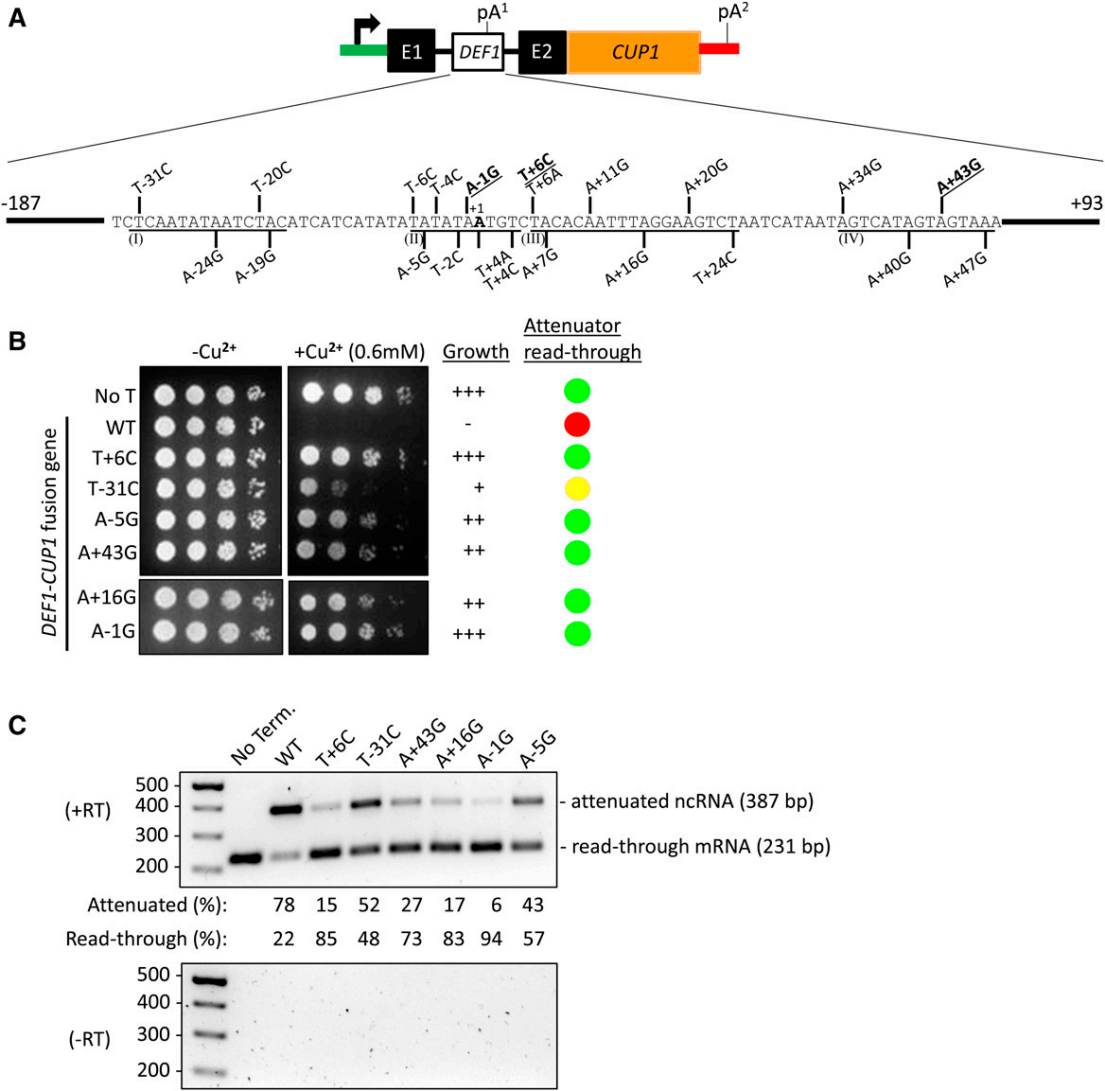
*Cis*-acting mutations result in Pol II read-through of the *DEF1* attenuator. (A) Schematic of *DEF1* attenuator sequences (boxed) that were fused to the *CUP1* reporter gene and used in a genetic selection for copper-resistant mutations. The individual point mutations resulting in a copper-resistant phenotype are numbered relative to +1 ATG start codon. Mutations that are underlined and in bold were the top 3 most frequent. Mutations were organized into regions I – IV based on clustering and similarity to pA site consensus elements. (B) Copper-resistant growth of yeast strains containing *DEF1-CUP1* reporter plated on -/+ copper plates after serial dilution. Higher levels of copper-resistance indicate higher levels of attenuator read-through (low-red, medium-yellow, high-green). (C) RNA analysis of *DEF1-CUP1* expression using RT-PCR (similar primers to Fig. 2C) to detect attenuated *vs.* full-length mRNA. (Top panel) The % attenuated *vs.* full-length RNA was determined by adding signal intensities together for both bands and determining the relative ratio. (Bottom panel) Negative control for RT-PCR reaction in which reverse transcriptase enzyme was withheld from the reaction.

In order to define the *cis*-acting sequence elements that promote *DEF1* attenuator recognition, we randomly mutagenized the *DEF1-CUP1* reporter and utilized a genetic selection to identify copper resistant colonies. We identified 22 attenuator point mutations within a 78 bp region (-31 to +47 relative to *DEF1* +1 ATG start codon) (Figure 6A). The most frequently identified mutants were A-1G (n = 8), T+6C (n = 5), A+43G (n = 3), A+40G (n = 2), and T-20C (n = 2). The remaining mutants were each identified once in the genetic selection. To compare the relative level of copper resistance, we conducted growth assays for six of the mutants and analyzed the ratio of attenuated:read-through transcripts via RT-PCR. The spot test and RT-PCR assays exhibited strong agreement with respect to attenuator activity. The WT *DEF1* attenuator resulted in no growth (-) on 0.6 mM copper and 78% usage of the attenuator (Figure 6B, C). The A-1G and T+6 mutants were most defective for attenuator recognition, resulting in strong copper-resistant growth (+++) and decreased attenuator usage (6–15%). The A+43G, A+16G, and A-5G mutants were of intermediate strength, and the T-31C mutant had the weakest effect of the mutants tested.

We arranged the mutants into four regions (I-IV) based on their clustering pattern and similarity to known 3′-end processing elements (Figure 6A) (Tian and Graber 2011). The most commonly used *DEF1* pA^1^ site in our previously reported RNA-Seq data was A+43 (Graber *et al.* 2013). Our identification of A+43G as a read-through mutant validates our genetic approach and defines region IV as a putative cleavage site (CS). Region II of the *DEF1* attenuator contains a consensus match to the yeast efficiency element (EE), and 5/22 unique DNA point mutations targeted the TATATA sequence. The mutations within the proposed EE include A-1G, which was the strongest and most-commonly identified read-through mutant. In yeast, the UAUAUA element serves as an RNA binding site for Hrp1 (Chen and Hyman 1998; Valentini *et al.* 1999). Based on its position between the EE and CS, region III of the *DEF1* attenuator is likely to contain a positioning element (PE). Region III contains a partial match (AATTTA) to the consensus PE (AATAAA), and the A+11G and A+16G mutants alter this proposed PE. The PE serves as an RNA-binding site for Rna15 (Gross and Moore 2001). The mutations we have identified in the EE and PE are consistent with *hrp1-1* and *rna15-58* mutants being defective for *DEF1* attenuator recognition (Figure 3G, 4B).

### HRP1 overexpression suppresses read-through defects of cis-acting DEF1 attenuator mutants

**Figure 7:**
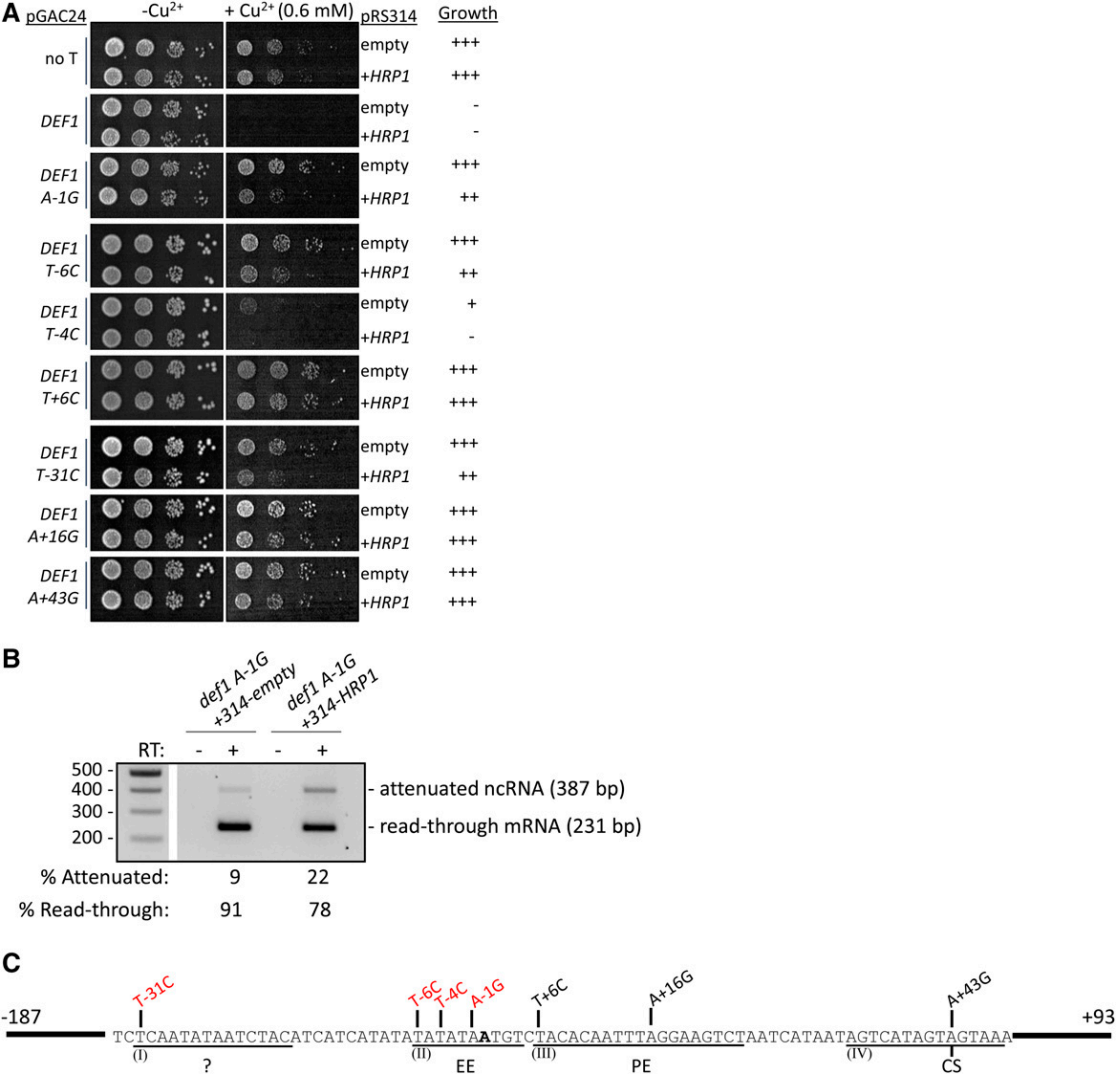
Hrp1 overexpression partially restores recognition of *cis*-acting *DEF1* attenuator mutants. (A) Reporter strains containing WT or mutant *DEF1-CUP1* reporters were transformed with an empty vector (pRS314) or a plasmid containing *HRP1 (*pRS314-*HRP1)* and grown on –Leu/Trp copper plates to assess *CUP1* expression. (B) Total RNA was collected from indicated strains grown at 30°C, and attenuated and read-through mRNAs were detected via RT-PCR. (C) Summary of genetic interactions between hyperactive *HRP1* allele and *cis*-acting *DEF1* attenuator mutants. The copper-sensitivity of mutants indicated in red was partially suppressed by *HRP1* overexpression, consistent with improved attenuator recognition and Hrp1-binding at RNA regions I and II.

Our identification of a pA site efficiency element (EE) and our characterization of the *hrp1-1* mutant suggested that Hrp1 may recognize the *DEF1* attenuator. To test whether Hrp1 binds to the *DEF1* EE or other regions of the attenuator, we overexpressed *HRP1* by transforming *cis*-acting mutant strains with a plasmid version of *HRP1* in addition to the chromosome. We chose a low-copy (CEN) *HRP1* plasmid since we have previously observed that high-copy *HRP1* expression from a 2μ plasmid is toxic (data not shown). We predicted that *HRP1* overexpression would enhance attenuator recognition, reduce *CUP1* reporter expression, and therefore increase copper sensitivity. As expected, the *CUP1* reporter lacking a terminator (No Term.) was copper-resistant, the wild-type *DEF1-CUP1* reporter was copper-sensitive, and *cis*-acting attenuator mutants conferred various degrees of copper resistance in the presence of the pRS314 empty vector control (Figure 7A). The A-1G, T-6C, and T-4C mutants were more copper-sensitive with *HRP1* overexpression (pRS314-*HRP1*) than an empty vector control, consistent with improved binding of Hrp1 to the mutant EE of region II. Surprisingly, the T-31C mutant was also more copper-sensitive with *HRP1* overexpression, suggesting that Hrp1 binds region I and/or that region I influences Hrp1 binding at region II. The effect of *HRP1* overexpression was allele-specific, with little-to-no genetic interaction observed between pRS314-*HRP1* and the *DEF1* attenuator mutants T+6C, A+16G, or A+43G in regions III and IV.

To confirm that increased copper-sensitivity was due to enhanced Pol II termination, we analyzed the ratio of attenuated:read-through transcripts by RT-PCR. As expected for enhanced attenuator recognition, *HRP1* overexpression increased attenuated RNA ∼twofold, resulting in less read-through mRNA compared to the empty vector control (Figure 7B). Taken together, our genetic analysis of the *DEF1* attenuator indicates that region II contains the EE and region IV contains the CS (Figure 7C). Region III is likely to contain the PE, and the role of region I is unclear but its activity is influenced by Hrp1.

### Attenuator mutant results in DEF1 overexpression and exacerbates toxicity of constitutively active Def1

**Figure 8:**
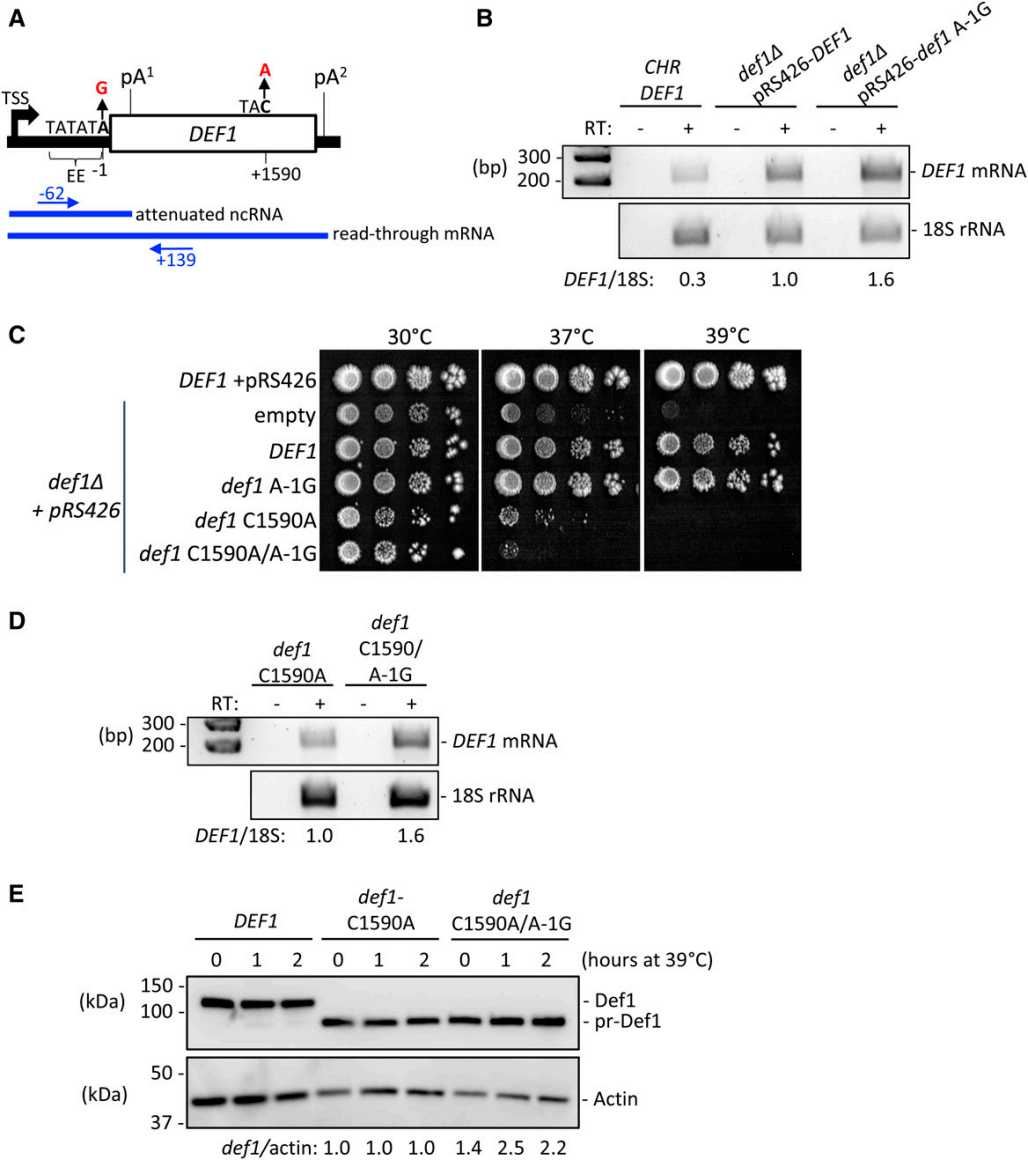
The *def1* A-1G attenuator mutant increases Def1 mRNA and protein and reduces cell viability when overexpressing pr-Def1. (A) Schematic of the *DEF1* gene (not to scale). The relevant pA sites, efficiency element (EE), mutations (A-1G, C1590A), and RT-PCR primers are indicated. (B) Yeast strains containing chromosomal (CHR) *DEF1* or *def1*Δ were transformed with empty vector (pRS426), WT *DEF1* (pRS426-*DEF1*), or mutant (pRS426-*DEF1*, *A-1G*) plasmids. Total RNA was isolated, and Def1 read-through mRNA was detected via RT-PCR (using blue primers indicated in (A). The signal intensity of the *DEF1* mRNA bands was normalized to the 18S loading control and then the *def1*Δ pRS426-*DEF1* sample. RT: Reverse Transcriptase. (C) Yeast strains containing WT chromosomal *DEF1* or *def1*Δ were transformed with empty vector (pRS426), WT *DEF1* (pRS426-*DEF1*), attenuator mutant (pRS426-*DEF1*, *A-1G*), pr-Def1 mutant (pRS426-*DEF1-C1590A*), or double mutant plasmids. Strains were spotted on -Ura plates and growth was assessed after 1 week at the indicated temperatures. (D) Total RNA was collected from strains in (C) containing *def1* mutants C1590A and C1590A/A-1G in a *def1*Δ strain grown at 30°C. *DEF1* read-through mRNA was detected via RT-PCR and quantified as in (B). (E) Western blot of extracts from strains in (C) following growth at 30°C and a temperature shift to 39°C for 0, 1, or 2 hr. Def1 protein levels were normalized to the actin loading control, and signal from *def1* C1590A/A-1G was normalized to def1 C1590A at consistent time points.

Thus far, our characterization of the *DEF1* attenuator utilized a reporter gene construct, with the terminator positioned within an *ACT1* intron and under transcriptional control of a constitutive *TDH3* promoter. To study the *DEF1* attenuator in a more natural context, we cloned the full-length *DEF1* gene (promoter, 5′-UTR, ORF, 3′-UTR) into a high-copy 2μ plasmid (pRS426) and transformed it into a *def1Δ* strain, where the plasmid was the sole source of *DEF1*. We designed measured *DEF1* attenuator activity based on the level of mRNA accumulation, using primers that amplified read-through RT-PCR products extending beyond pA^1^ (Figure 8A). We observed a ∼1.6 increase in read-through mRNA in the A-1G attenuator mutant compared to wild-type, indicating that the natural *DEF1* attenuator functions to suppress transcription (Figure 8B).

Given that Def1 protein expression exhibits post-translational regulation (Wilson *et al.* 2013; David 2013), we sought to establish biological significance for our observed transcriptional regulation. To test the biological significance of the *DEF1* attenuator in isolation from post-translational regulation we created a *def1* C1590A allele, which introduces a mutation (C1590A) that results in a premature stop codon (TAC→TAA) (Figure 8A). The truncated version of Def1 (pr-Def1) produced from *def1* C1590A mimics a UV-dependent processing event (Wilson *et al.* 2013). At elevated temperatures, the pr-Def1 protein becomes activated via nuclear localization, triggers Pol II ubiquitination and degradation, and is toxic to cells. We predicted that attenuator mutations would elevate levels of *DEF1* mRNA and pr-Def1 protein, resulting in greater cell toxicity.

We analyzed the growth of yeast strains containing an attenuator mutation (*def1* A-1G) in the context of pr-Def1 expression (*def1* C1590A). At the permissive temperature of 30°, all strains grew at a similar rate and density (Figure 8C). As expected, the *def1* C1590A mutation resulted in heat-sensitivity at 37° and 39°, similar to the *def1Δ* strain with an empty vector (Wilson *et al.* 2013). Transformation of *def1Δ* with pRS426-*DEF1* restored growth to wild-type levels, consistent with full complementation by the plasmid-based *DEF1* allele. The *def1* A-1G attenuator mutant grew similarly to wild-type, presumably because transcriptional overexpression was masked by post-translational protein control. Interestingly, the *def1* A-1G/C1590A double mutant exhibited a synthetic sick phenotype at the non-permissive temperature of 37°. To better quantify this genetic interaction, we determined the growth rate of strains in liquid culture. The pRS426-*DEF1* strain had a doubling time of 2.2 hr and 2.5 hr, at 30° and 39° respectively. In contrast, the *def1* C1590A mutant had a doubling time of 3.0 hr and 5.3 hr, consistent with a heat-sensitive defect. The *def1* A-1G/C1590A mutant had a doubling time of 3.0 hr and 7.9 hr, exhibiting a more severe growth defect at 39° than the *def1* C1590A single mutant.

To confirm that *def1 A-1G* enhanced *def1 C1590A* toxicity due to pr-Def1 overexpression, we quantified mRNA and protein levels. The *def1* A-1G mutation increased mRNA expression by 1.6 compared to *def1* C1590 alone (Figure 8D). This mRNA overexpression correlated with ∼twofold more Def1 protein in *def1* A-1G/C1590A compared to *def1* C1590A (Figure 8E). Overall, these data are consistent with Pol II attenuation contributing to *DEF1* regulation in yeast and serving an important biological function.

## DISCUSSION

Termination is one of the least understood aspects of Pol II transcription, and a further knowledge gap exists for regulation by premature termination (attenuation). In this study we conducted a thorough characterization of an attenuator in the *DEF1* DNA repair gene, which bears unique features unseen in previous attenuator studies. The *DEF1* attenuator relies on a hybrid of termination factors for efficient recognition, with a bias for the CPF-CF pathway *vs.* the NNS pathway. We have identified nine termination factors and four RNA sequence elements that contribute to attenuator activity, including the Hrp1 RNA-binding protein, a putative pA site efficiency element, and the region around the pA site itself. Furthermore, we have shown that disruption of the attenuator is biologically significant, supporting a new role for transcription attenuation in regulating a DNA damage response gene.

### The DEF1 attenuator exhibits a unique hybrid attenuator biased toward CPF-CF termination

Our mutational analysis indicates that *DEF1* attenuator recognition involves both CPF-CF and NNS termination pathways, but it is more reliant on CPF-CF recognizing a traditional pA site (Figure 6A; regions II, III, and IV). The importance of *DEF1* attenuator region I is unclear, but it may influence Hrp1 binding to region II, perhaps by forming a secondary structure. The *DEF1* attenuator exhibits Sen1-dependence but little-to-no dependence on Nrd1 or Nab3 despite sequence similarity to consensus Nrd1 (GUAA, GUAG) and Nab3 RNA-binding sites (UCUU) (Steinmetz and Brow 1998; Carroll *et al.* 2004; Creamer *et al.* 2011; Porrua *et al.* 2012). The very limited role for Nrd1 and Nab3 at the *DEF1* attenuator is a contrast to what has been observed for most other attenuators, including *NRD1*, *IMD2*, *URA2*, *FKS2*, *CLN3*, *GPH1*, and *GLT1*, which exhibit strong dependence on Nrd1, Nab3, or both (Arigo *et al.* 2006; Jenks *et al.* 2008; Kuehner and Brow 2008; Thiebaut *et al.* 2008; Kim and Levin 2011; Darby *et al.* 2012; Chen *et al.* 2017; Merran and Corden 2017). The lack of Nrd1/Nab3 involvement that we observe is consistent with *DEF1* expression levels not increasing upon Nrd1/Nab3 depletion and Nrd1/Nab3 failing to crosslink to *DEF1* during *in-vivo* crosslinking studies (Jamonnak *et al.* 2011; Merran and Corden 2017).

*DEF1* attenuator recognition is dependent on CFI component Hrp1 rather than Nrd1 or Nab3, which is somewhat surprising given its promoter-proximal location. In the reporter system, the TSS to EE distance is 365 bp, but this distance is only ∼100 bp in the natural *DEF1* context, and we observe a termination defect in both cases with the *def1* A-1G mutant. At individual genes, Hrp1 has been shown to crosslink to coding regions ∼twofold better than the promoter, and in some cases Hrp1 shows the strongest occupancy at the pA site near the 3′-ends of genes (Komarnitsky *et al.* 2000; Mayer *et al.* 2010). However, Hrp1 is proposed to bind within its own 5′-UTR as a mean of autoregulation, suggesting that it can act near gene promoters (Steinmetz *et al.* 2006b; Kuehner and Brow 2008; Chen *et al.* 2017). Furthermore, genome-wide crosslinking indicated that the majority of Hrp1 is bound to promoter-proximal regions of mRNAs (Tuck and Tollervey 2013), and transcriptome analysis in an *hrp1* mutant revealed sn/snoRNA termination defects on approximately one-third of the sn/snoRNA genes (Chen *et al.* 2017). Overall, these data indicate that Hrp1 acts more generally as both a CPF-CF and an NNS termination factor.

Another unique feature of the *DEF1* attenuator is its seeming lack of dependence on Pcf11. None of the *pcf11* mutants altered *DEF1* attenuator recognition despite the termination defects they exhibited for the *SNR13* control terminator. At other gene targets, the *pcf11-2* mutation impairs mRNA cleavage, the *pcf11-13* mutation impairs Pol II CTD-binding, and *pcf11-9* impairs both cleavage and Pol II CTD-binding. Accordingly, these mutants disrupt CPF-CF, NNS, or both termination pathways (Amrani *et al.* 1997; Sadowski *et al.* 2003; Kim *et al.* 2006; Grzechnik *et al.* 2015). The termination defect of *pcf11-13* corresponds with failed release of Nrd1, reduced Ser2 CTD phosphorylation, and lack of Sen1 recruitment (Grzechnik *et al.* 2015). Seemingly the *DEF1* attenuator does not require this Pcf11 function to elicit termination, perhaps because the Nrd1 and Nab3 proteins are not required either.

We have confirmed that *DEF1* attenuator recognition requires Hrp1, Rna14, Rna15, Ssu72, Ctk1, Glc7, Cft2, Paf1, and Sen1. Like Hrp1, several of these proteins may contribute to *DEF1* pA^1^ site recognition. Rna15 and Rna14 are also members of CFI, and while Rna15 recognizes pA site positioning elements, Rna14 is capable of bridging Rna15 and Hrp1 in a CFI complex (Gross and Moore 2001; Barnwal *et al.* 2012). Cft2 is a component of the core CPF that binds the *CYC1* pA site *in vitro* and crosslinks near pA sites *in vivo*, as well as interacting with the Pol II CTD (Dichtl and Keller 2001; Kyburz *et al.* 2003; Baejen *et al.* 2014).

In lieu of direct *DEF1* pA^1^ site recognition, some proteins may promote recruitment of termination factors. Ctk1 is a kinase that phosphorylates Ser2 residues of the Pol II CTD, an event that can occur relatively early in transcription and perhaps lead to promoter-proximal termination via recruitment of Sen1 (Mayer *et al.* 2010; Chinchilla *et al.* 2012; Lenstra *et al.* 2013). Glc7 and Ssu72 are components of the APT sub-complex of CPF, also termed the phosphatase module, which are required for termination of both mRNA and noncoding RNA genes (Mayfield *et al.* 2016; Casañal *et al.* 2017). Removal of Ser5-P from the Pol II CTD occurs via Ssu72, which is present at both the 3′-end of genes as well as promoter regions (Singh and Hampsey 2007; Zhang *et al.* 2012). Defects in Ssu72 increase Ser5-P, which may disrupt Ser2-P accumulation and Sen1:Pol II association. Glc7 promotes the removal of Tyr1-P from the Pol II CTD, and failure to remove Tyr1-P prevents recruitment of termination factors, including Nrd1, Pcf11, and Rtt103 (Mayer *et al.* 2012; Schreieck *et al.*, 2014). Paf1 has been implicated in both CPF-CF and NNS termination, and *paf1* mutants exhibit altered histone modification and reduced Ser2-P levels (Sheldon *et al.* 2005; Nordick *et al.* 2008; Tomson *et al.* 2011; Terzi *et al.* 2011). There is also precedence for Paf1 helping to recruit CPF to Ser5-P CTD, consistent with Paf1:Pol II enrichment on transcripts containing CPF proteins Cft2 and Mpe1 (Nordick *et al.* 2008; Fischl *et al.* 2017).

Sen1 may contribute most directly to termination at the *DEF1* attenuator via ATP-dependent RNA translocation and destabilization of paused Pol II, perhaps via melting of the RNA:DNA active site hybrid (Kim *et al.* 1999; Porrua and Libri 2013; Martin-Tumasz and Brow 2015; Han *et al.* 2017; Leonaitė *et al.* 2017). Given the limited role of Nrd1 and Nab3 in *DEF1* attenuation, Sen1 recruitment could occur instead through CPF component Glc7 or direct interaction with Ser2-P CTD, or connecting to CFI protein Hrp1 via CFI-CPF cross-factor interactions (Preker *et al.* 1995; Ohnacker *et al.* 2000; Kyburz *et al.* 2003; Holbein *et al.* 2011; Ghazy *et al.* 2012; Chinchilla *et al.* 2012).

### Regulation of DEF1 by dual transcriptional and post-translational mechanisms

Our evidence suggests that Pol II transcriptional attenuation contributes to biologically meaningful *DEF1* regulation in addition to a previously described post-translational mechanism (Wilson *et al.* 2013). Constitutive expression of a truncated protein that mimics Def1 activation is lethal to cells (Wilson *et al.* 2013), consistent with tight control of Def1 expression being vital for cell survival. *DEF1* expression and function contributes to several biological processes, including rescue of stalled Pol II, nucleotide excision repair, telomere maintenance, translesion synthesis, and Pol II initiation (Woudstra *et al.* 2002; Chen *et al.* 2005; Daraba *et al.* 2014; Damodaren *et al.* 2017). In addition, Def1 contributes resistance to DNA damage stress, salt stress, and heat shock stress (Woudstra *et al.* 2002; Vanacloig-Pedros *et al.* 2015; Damodaren *et al.* 2017). Given that both Def1 depletion and overexpression can be lethal, cells likely evolved multiple regulatory mechanisms to maintain *DEF1* homeostasis. An advantage of Pol II attenuator read-through *vs.* upregulation of initiation is that it could provide a more rapid response to an environmental stressor, akin to release of paused Pol II (Adelman and Lis 2012).

There is limited evidence to explain the mechanism by which Pol II attenuator read-through occurs for stress response genes. In the case of *IMD2*, depletion of the guanine nucleotide pool causes Pol II to shift from an upstream guanine TSS to a downstream adenine TSS, bypassing an NNS-dependent attenuator and allowing synthesis of full-length mRNA (Steinmetz *et al.* 2006b; Jenks *et al.* 2008; Kuehner and Brow 2008). The Levin and Manley labs have described an alternative mechanism for regulation of the *FKS2* attenuator. Cell wall damage activates the MAP kinase Mpk1, which associates with Pol II and Paf1, phosphorylates Tyr1 of the Pol II CTD, restricts Nrd1 recruitment, and allows Pol II to bypass an attenuator and terminate at a downstream pA site (Kim and Levin 2011; Yurko *et al.* 2017). We have identified several characteristics that are unique to *DEF1* *vs.* *IMD2* and *FKS2* attenuators, perhaps reflecting a novel mechanism for Pol II recognition and read-through. *DEF1* will be a useful model to expand our understanding of the signaling mechanisms that modulate Pol II termination, particularly during stress adaptation to DNA damage. It will also be of interest to explore connections between yeast Pol II attenuator function and alternative polyadenylation (APA) in metazoans. Splicing plays a dominant role in usage of promoter-proximal intronic pA sites, but upstream exonic pA sites remain relatively unexplored (Luo *et al.* 2013; Tian and Manley 2017).
